# Novel Somatic Mutations to PI3K Pathway Genes in Metastatic Melanoma

**DOI:** 10.1371/journal.pone.0043369

**Published:** 2012-08-17

**Authors:** Austin Y. Shull, Alicia Latham-Schwark, Poornema Ramasamy, Kristin Leskoske, Dora Oroian, Marc R. Birtwistle, Phillip J. Buckhaults

**Affiliations:** 1 Georgia Health Sciences University Cancer Center, Georgia Health Sciences University, Augusta, Georgia, United States of America; 2 College of Medicine, Medical University of South Carolina, Charleston, South Carolina, United States of America; 3 Department of Molecular and Cell Biology, University of California, Berkeley, California, United States of America; Faculdade de Medicina, Universidade de São Paulo, Brazil

## Abstract

**Background:**

BRAF^V600^ inhibitors have offered a new gateway for better treatment of metastatic melanoma. However, the overall efficacy of BRAF^V600^ inhibitors has been lower than expected in clinical trials, and many patients have shown resistance to the drug’s effect. We hypothesized that somatic mutations in the Phosphoinositide 3-Kinase (PI3K) pathway, which promotes proliferation and survival, may coincide with BRAF^V600^ mutations and contribute to chemotherapeutic resistance.

**Methods:**

We performed a somatic mutation profiling study using the 454 FLX pyrosequencing platform in order to identify candidate cancer genes within the MAPK and PI3K pathways of melanoma patients. Somatic mutations of theses candidate cancer genes were then confirmed using Sanger sequencing.

**Results:**

As expected, BRAF^V600^ mutations were seen in 51% of the melanomas, whereas NRAS mutations were seen in 19% of the melanomas. However, PI3K pathway mutations, though more heterogeneous, were present in 41% of the melanoma, with PTEN being the highest mutated PI3K gene in melanomas (22%). Interestingly, several novel PI3K pathway mutations were discovered in MTOR, IRS4, PIK3R1, PIK3R4, PIK3R5, and NFKB1. PI3K pathway mutations co-occurred with BRAF^V600^ mutations in 17% of the tumors and co-occurred with 9% of NRAS mutant tumors, implying cooperativity between these pathways in terms of melanoma progression.

**Conclusions:**

These novel PI3K pathway somatic mutations could provide alternative survival and proliferative pathways for metastatic melanoma cells. They therefore may be potential chemotherapeutic targets for melanoma patients who exhibit resistance to BRAF^V600^ inhibitors.

## Introduction

Over the past five years, considerable progress has been made in the clinical treatment options for metastatic melanoma. Small molecule inhibitors targeting active kinases have generated remarkable clinical responses in a high proportion of melanoma patients [1]. However, not all patients respond to these agents, and resistant relapses are beginning to be documented [2]. The National Cancer Institute estimates that 1 out of 51 Americans will be diagnosed with melanoma during some point in their life, underscoring the importance of augmenting these successes with additional therapies targeting alternate signaling pathways [http://seer.cancer.gov/statfacts/html/melan.html].

The Mitogen Activated Protein Kinase (MAPK) pathway, when dysregulated, is an important driver of several cancer types, including metastatic melanoma [3]. The canonical MAPK pathway consists of the Ras family proteins (K-ras, H-ras, N-ras), Raf protein kinases (A-raf, B-raf, C-raf), mitogen and extracellular-regulated protein kinase kinase (Mek 1, Mek 2), and extracellular signal-related kinase (Erk 1, Erk 2). When GTP-bound, activated Ras recruits Raf to the plasma membrane where its kinase function is activated. This enables Raf to phosphorylate Mek, which in turn phosphorylates and activates Erk [4], [5]. Erk phosphorylates many different substrates, thus affecting multiple cellular responses including cell proliferation, senescence, and differentiation [5]. Melanomas often contain improperly activated MAPK signaling, which results in continuous cell proliferation and survival.

Activating somatic mutations in the MAPK pathway have been very popular targets for the development of specialized targeted chemotherapeutics. Therapeutic drugs have been developed that selectively target the Ras family of proteins, key upstream activators of MAPK proteins, which are mutated in one-third of all human cancers [6]. Recently, the BRAF oncogene has been identified as an important upstream kinase for driving MAPK signaling in melanoma [7]. Newly developed BRAF inhibitory drugs selectively target B-raf proteins that contain a somatic mutation in exon 15, affecting amino-acid position 600. The most common mutations consist of a single-base missense substitution that changes the amino acid valine to a glutamate (BRAF^V600E^) or to a lysine (BRAF^V600K^). This mutation hotspot is altered in over 50% of all metastatic melanoma tumors [7], [8], [9].

Recent clinical studies have shown that most BRAF mutant positive melanoma patients treated with mutant-BRAF^V600^ specific inhibitors, like vemurafenib, initially have a dramatic clinical response. However these tumors almost universally quickly evolve resistance to the drug, leading to clinical relapse [10]. In spite of these relapses, targeted BRAF^V600^ inhibitors still perform better than sorafenib, a BRAF inhibitor that targets both wild-type and mutant kinases [11]. Similar results have been reported for other MAPK monotherapies [6].

New mechanistic studies indicate that sustained cancer proliferation occurs through dysregulation of the Phosphoinositide 3-Kinase (PI3K) pathway acting in complementary fashion to the mutated MAPK pathway [12]. Key components of the PI3K signaling cascade are the p110á catalytic subunit (PIK3CA), the Phosphatase and Tensin homolog (PTEN) tumor suppressor, the downstream effector serine/threonine kinases (Akt), and the mammalian target of rapamycin (mTOR). Twenty-six percent of breast cancers and 12% of large intestinal cancers have somatic mutations to PIK3CA [13], making mutant PIK3CA a popular target for the development of chemotherapeutic agents [14]. When PIK3CA is mutated, Akt signaling is stimulated, increasing cell proliferation and disease metastasis. In normal cell signaling, the tumor suppressor PTEN antagonizes p110á signaling due to its encoded phosphatase activity [15]. However, when PTEN is inactivated through somatic mutations, its negative regulatory function is abolished, allowing p110á to activate Akt in an unchecked manner. Activated Akt can stimulate cell proliferation by activating downstream effectors like mTOR and can inhibit apoptosis by mechanisms such as MDM2-mediated p53 degradation and Bcl-2-associated death promoter (BAD) phosphorylation [16], [17], [18], [19].

It is thought that when the MAPK and PI3K pathway are dysregulated, both will work synergistically to increase cellular proliferation, survival and disease progression [8], [10]. One possible explanation involves the well-known strong negative feedback property of the MAPK pathway [20], [21]. For example, when B-raf becomes constitutively active, strong negative feedback from Erk works to mitigate this increased mitogenic signaling. With this understanding, parallel mutations to the PI3K pathway may help offset these negative-feedback induced losses in the MAPK pathway. Moreover, increased signaling through the MAPK pathway is associated with increased apoptotic signaling through the MST2 pathway [22], [23], [24]. Thus, parallel PI3K pathway mutations may also help increase survival signaling to offset such apoptotic signaling.

Many established nodal points in the PI3K pathway (i.e. PIK3CA, AKT, PTEN) have been linked to melanoma progression. Therefore, most treatment strategies are focused on these well-established altered regulators, though with limited success. However, it may prove beneficial to explore the possibility that other PI3K pathway members are frequently mutated and therefore targets for effective therapies. Here, we used 454 FLX amplicon sequencing and performed gene mutation profiling of the MAPK and PI3K pathways in melanoma to determine if novel somatic mutations occurred in a mutually exclusive or complementary fashion with BRAF^V600^ mutations. By identifying the heterogeneity of MAPK and PI3K mutations in melanoma tumors, we can generate new hypotheses for how to inhibit melanoma progression through novel combinations of chemotherapeutics.

## Materials and Methods

### Primer Selection and Testing

This study was conducted in three phases: a Discovery phase, in which all of the 31 candidate genes were sequenced in 24 “discovery” tumors, and two Prevalence phases, which further analyzed the genes mutated in the Discovery phase in an additional 44 tumors (Table S1) [25]. Exon-flanking primer pairs were designed using Primer 3 (http://frodo.wi.mit.edu/primer3/) for a total of 566 unique amplicons covered all coding exons of the 31 candidate genes existing in either the PI3K or MAPK pathway (Table 1). Primers were 5¢-tagged with 454A (GCCTCCCTCGCGCCATCAG-Forward Exon Primer) and 454B (GCCTTGCCAGCCCGCTCAG-Reverse Exon Primer) linker sequences. All primer pairs were tested using the KAPA HiFi HotStart PCR kit (Kapa Biosystems, Capetown South Africa) in a 25 µl reaction volume containing 1 µl of 4 ng/µl normal human genomic DNA template, 5 µl 5X HiFi Reaction Buffer, 0.75 µl of 10 µM KAPA dNTP mix, 0.5 µl KAPA HiFi HotStart DNA Polymerase, 14.75 µl PCR grade nuclease-free H_2_O, and 3 µl forward/reverse primer mix (5 µM each primer). The following touchdown thermocycling protocol was utilized: 98°C for 2 min, one cycle; 98°C for 20 sec, 64°C for 10 sec, and 70°C for 30 sec, three cycles; 98°C for 20 sec, 61°C for 10 sec, and 70°C for 30 sec, three cycles; 98°C for 20 sec, 58°C for 10 sec, and 70°C for 30 sec, three cycles; 98°C for 20 sec, 57°C for 10 sec, and 70°C for 30 sec, 50 cycles, 70°C for 5 min, one cycle. PCR products were then analyzed by agarose gel electrophoresis. These conditions generated a success rate of 85%. Primers that failed to produce the expected PCR products were redesigned with Primer3 and the above process was repeated. Re-designed primers had a success rate of 50%, leading to an overall coverage for all amplicons of 95%. Primer sequences are available from the authors upon request.

**Table 1:** List of PI3K and MAPK pathway genes sequenced.

Gene	Pathway
IRS2	PI3K
IRS4	PI3K
PIK3R1	PI3K
PIK3R4	PI3K
PIK3R5	PI3K
PIK3CA	PI3K
PTEN	PI3K
NFKB1	PI3K
CHUK/IKK	PI3K
RPS6KA2	PI3K
RPS6KB1	PI3K
RHEB	PI3K
AKT1	PI3K
AKT2	PI3K
AKT3	PI3K
PDPK1	PI3K
SHIP1	PI3K
FOXO1	PI3K
FOXO3	PI3K
FOXO4	PI3K
PP2A	PI3K
TSC1	PI3K
TSC2	PI3K
BRAF	MAPK
NRAS	MAPK
MAPK1	MAPK
MAPK3	MAPK
EGFR	MAPK
KIT	MAPK
MTOR	PI3K
FBXW7	MAPK

### Quantification of Samples

The samples utilized for this study are from 68 melanoma tumor cell lines established by the Surgical Branch of the National Cancer Institute. The correlating normal DNAs were obtained from patient-matched peripheral blood mononuclear cells (Table S1) [25]. The concentrations of all tumor gDNA samples were determined by Real-Time PCR using iQ™ SYBR® Green Supermix (Bio-Rad) and Primers directed to conserved regions of Long Intersperse Nuclear Elements (LINE) in a 25 µl reaction. 20-fold dilutions of each tumor gDNA sample were used in the reaction volume containing 12.5 µl SYBR® Green Supermix, 6.5 µl PCR grade nuclease-free H_2_O, 5 µl forward/reverse LINE primer mix (5 µM), and 1 µl of the diluted template. Each sample was quantified in triplicate in conjunction with triplicates of known DNA standards. The sequences of the LINE primers were LINEF (AAAGCCGCTCAACTACATGG) and LINER (CTCTATTTCCTTCAGTTCTGCTC).

### Normalized Tumor Pool Preparation

Following LINE-based quantification, three concentration-normalized gDNA tumor pools were produced using 24 tumor samples for the Discovery phase, 22 for the Prevalence 1 phase, and 22 for the Prevalence 2 phase. These pools were developed by aliquoting the volume of each stock tumor to contain 180 ng of DNA, resulting in a tumor pool that included 1.5 ng/µl of gDNA per tumor sample.

### PCR Amplification and 454FLX Amplicon Sequencing of Normalized Tumor Pool

Amplicons were prepared using tumor pools as DNA templates, resulting in the parallel amplification of all variant alleles in each pool. Each reaction contained a total of 75 ng of genomic DNA (approximately 3 ng from each of the 24 tumors). PCR products were analyzed by agarose gel electrophoresis and then were stored at 0°C until purification. All PCR products were purified using the AmPure® Purification System (Agencourt, Beverly MA), according to the manufacturer’s recommendations. Briefly, 18 µl AmPure® beads were added to 21 µl raw PCR products, and the manufacturer’s protocol was followed. The purified PCR products were eluted in 100 µl TE buffer (pH 8)**.** The individual purified PCR products were then quantified on a Fluoroskan Ascent Microplate Fluorometer (Thermo Scientific) using Quant-iT™ PicoGreen® dsDNA kit (Invitrogen, San Diego, CA). Equal amounts of each amplicon (50 ng) were combined together, and the resulting amplicon pools were sequenced using the 454 FLX (Roche, Basel, Switzerland) platform from the University of South Carolina Environmental Genomics Core Facility. Two runs were performed for each tumor pool using ∼250 amplicons per run.

### Variant Detection

454 sequencing reads (FASTQ) were assembled to the hg19/b37 human genome reference sequence using CLC Genomics Workbench 4 (CLC Bio, Aarhus Denmark). Single nucleotide variants (SNVs) were identified with a cutoff of 1% minor allele frequency, and a minimum of 10 variant reads. Intronic variants were discarded, and all exonic variants were analyzed to determine effects on the resulting coding sequence (non-synonymous or synonymous). Variants were annotated with dbSNP, 1000 Genomes and COSMIC databases, and those matching 1000 Genomes, HapMap, or 1000 Exomes variants were classified as germline mutations, whereas those not contained within any of these databases were classified as possible somatic mutations. Genes were rank-ordered in likelihood of being a cancer driver gene by their overall rates of somatic mutations and their ratios of non-synonymous/synonymous (NS/S) variants.

### Variation Confirmation and Tumor/Normal Pair Sequencing

In order to further confirm the nucleotide variations found by 454 FLX sequencing and identify the original tumor(s) from which the variants were derived, each amplicon containing a potential somatic variant was reamplified from the individual tumor gDNA samples used to make the pools, along with patient-matched normal gDNA samples. PCR amplification was performed with KAPA SYBR® FAST qPCR reagents and the following reaction conditions: 20 µl reaction containing 1 µl template DNA (∼3 ng), 7 µl PCR grade nuclease-free H_2_O, 10 µl KAPA SYBR® FAST qPCR Master Mix, and 2 µl forward/reverse primer mix (5 µM each). Reactions were conducted with the iCycler iQ™ Real Time PCR system (Bio-Rad) using the following cycling conditions: 95°C for 2 min, one cycle, 94°C for 10 sec, 64°C for 10 sec, and 70°C for 30 sec, 3 cycles; 94°C for 10 sec, 61°C for 10 sec, and 70°C for 30 sec, 3 cycles; 94°C for 10 sec, 58°C for 10 sec, and 70°C for 30 sec, 3 cycles; 94°C for 10 sec, 55°C for 10 sec, and 70°C for 30 sec, 3 cycles. These amplification steps were followed by a final melting curve starting at 55°C and increasing in 0.5°C increments every 10 sec for 80 cycles. The SYBR green fluorescence data collected during the real-time PCR served to verify that PCR products were made from each tumor. (In several cases involving PTEN, the real-time PCR fluorescence data revealed homozygous deletion of multiple exons in tumor, but not matched normal DNA samples. Properly amplified products were then bi-directionally Sanger sequenced (Beckmann Coulter Genomics, Beverly MA) using the 454 Tag A and 454 Tag B linkers as sequencing primers.

Sequence data was aligned to the human reference sequence with CLC Genomics Workbench. All chromatograms produced by Sanger sequencing were manually inspected and compared to the reference sequence in order to determine which tumors contained the suspected variant, and if the variant was indeed a somatic mutation or a germline variant. Some PCR reactions failed to amplify the desired product from tumor pools, and were therefore not covered sufficiently by 454 sequence data. To ensure adequacy of gene coverage, such amplicons were therefore created individually using HiFi HotStart reagents and Sanger sequenced as individual samples.

## Results

### Comparison of Germline & Somatic Subsets Derived from 454 FLX Sequencing Platform

**Figure 1:**
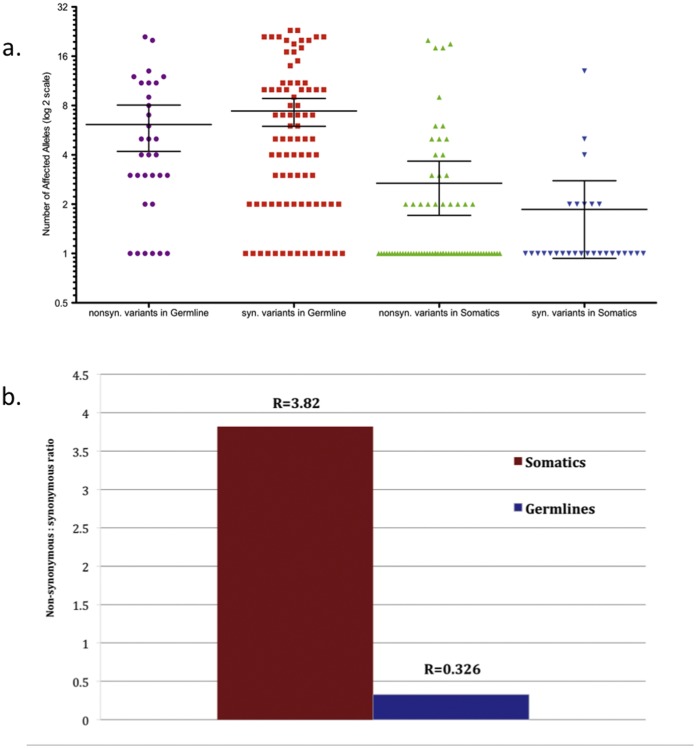
Distribution of allele frequencies of non-synonymous and synonymous germline and somatic variants in melanoma patients. (a) Allele frequencies of germline and somatic variants. The mean allele frequencies were: higher for germline variants (ns = 6.125, s = 7.412) than for the somatic variants, (ns = 2.685, s = 1.857). (b) Comparison of the ratios of non-synonymous to synonymous germline and somatic variants. Both were significantly different from the 2∶1 ratio expected by chance alone, but in opposite directions (somatic, binomial p = 1.15354×10^−5^; germline binomial p = 6.3953×10^−126^).

We identified a total of 769 germline and 241 somatic variants from the pooled 454 FLX sequencing of the 68 metastatic melanoma patients (Table S2). The concentration of each variant was proportional to the number of alleles affected in the total population. The germline variants were greater in number, and were highly enriched for synonymous mutations (mean = 6.13 NS: 7.41 S). In contrast, there were fewer somatic variants, and these variants were highly enriched for non-synonymous mutations. Notably, many of these somatic variants were private in nature between individual patients (mean = 2.68 NS: 1.86 S) (Figure 1a). For the germline variants, there were 189 non-synonymous variants and 580 synonymous variants, for a germline NS/S ratio of 0.326, which is significantly lower than the 2∶1 ratio expected by chance alone (binomial p-value = 6.395×10^−126^). This indicates that the subset of genes that were sequenced in this project is under strong negative selective pressure for alterations to the amino acid sequence during germline transmission. In contrast, we observed 191 non-synonymous somatic variants and 50 synonymous somatic variants, for a somatic NS/S ratio of 3.82, which is significantly higher than the 2∶1 ratio predicted by chance alone (binomial p-value = 1.154×10^−5^). This result indicates that the subset of genes sequenced in this project is under strong positive selective pressure for alterations to the amino acid sequence during melanoma progression. This large NS/S ratio difference highlights the opposing selective pressures experienced by these candidate cancer genes under the two different circumstances (Figure 1b).

### Rank Ordering of Cancer Candidate Genes

Our next goal was to prioritize possible cancer driver genes by quantifying the total number of non-synonymous mutations observed in each gene and comparing it to the number of non-synonymous mutations expected based on background mutation rates, the sizes of the respective coding sequences, and the total number of tumor genomes sequenced. Based on the number of synonymous mutations discovered within the sequenced amplicons, we calculated a background somatic mutation rate of 23.3 mutations per MB. This result is similar to that reported from whole exome sequencing (average over 14 patients, 11.2 mutations per MB) [26]. It is notable, however, that another study has reported substantial patient-to-patient variability (111 mutations/MB –3 mutations/MB, average = 30 mutations/MB) [27]. Because these background rates predict the occurrence of between 1 and 3 chance somatic mutations per gene to be present in our group of 68 patients, we considered any gene in our experiment that contained 2 or more somatic mutations as a possible candidate cancer gene. BRAF, PIK3R5, NFKB1, NRAS, IRS2, FOXO1, SHIP1, IRS4, PIK3CA, MTOR, PIK3R1, PIK3R4, and PTEN each had more than 1 somatic mutation and constitute reasonable candidate cancer driver genes.

**Figure 2:**
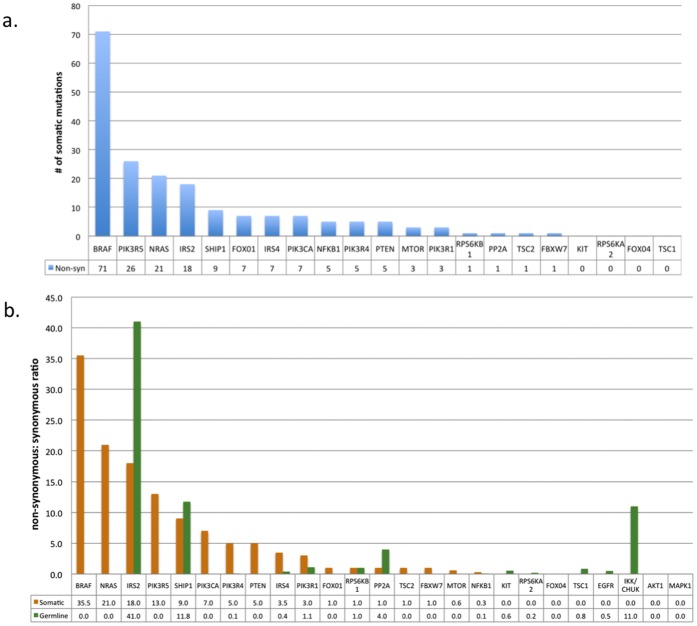
Counts and ratios of variants discovered in each sequenced gene. The absolute number of non-synonymous somatic variants for each gene is shown in (a) and the comparison of NS/S ratios for the germline and somatic variants of each gene is shown in (b). Blue shaded bars in (a) are non-synonymous mutations. The orange shaded bars are the NS/S ratios for the somatics variants, and the green shaded bars are the NS/S ratios for the germline variants.

We rank-ordered the resulting candidate genes based on the total number of non-synonymous somatic mutations found within the gene. A gene was considered a more likely cancer candidate gene if it possessed a high non-synonymous mutations (Figure 2a). As expected, we found the known melanoma drivers NRAS and BRAF to have the largest numbers of non-synonymous mutations in our candidate list. We also observed several novel candidate cancer genes, one of which was PIK3R5. This particular gene has not been implicated as a driver of melanoma; however, the overexpression of the associated catalytic p100ã subunit has been shown to induce sarcomagenesis through *in vitro* cell studies [28]. Other frequently mutated genes included SHIP1, IRS2, IRS4, PIK3CA, PIK3R4 and PTEN. NFKB1, MTOR, PIK3R1, and FOXO1, had fewer non-synonymous mutations, and a corresponding low non-synonymous/synonymous ratio, suggesting that these genes have a lower probability of being drivers, but still may warrant functional study.

A useful metric for rank-ordering genes by probability of being functionally important in cancer is to compare the NS/S ratio observed in both somatic and germline mutations (Figure 2b). We reason that genes that were most likely to be candidate drivers may have higher somatic NS/S ratios than germline NS/S ratios. This additional measurement reveals that IRS2 and SHIP1 had high levels of germline variants as well as somatic variants, placing them at lower priority for functional analysis.

### Combinations of Cancer Gene Mutations in Individual Melanoma Samples

**Figure 3:**
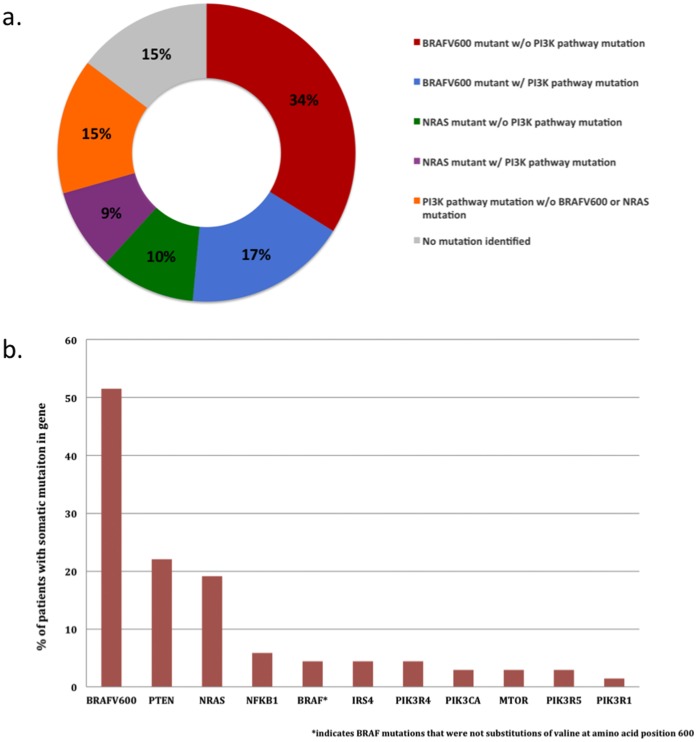
Distribution of melanoma patients according to pathway somatic mutations. (a) Percentages of melanomas that have: a BRAF^V600^ mutation without a PI3K pathway mutation, a BRAF^V600^ mutation with a PI3K pathway mutation, a NRAS mutation without a PI3K pathway mutation, a NRAS mutations with a PI3K pathway mutation, and a PI3K pathway mutation with wild-type BRAF^V600^ and NRAS. (b) Number of patients who carried the somatic mutations identified by Sanger sequencing.

To validate the mutations by an independent technology, and to determine which tumor in the pool contained the mutation, we Sanger sequenced the affected amplicons from each melanoma and matched normal sample. The results revealed that 35 of our 68 tumor samples contained a BRAF^V600^ mutation, and 13 of the 68 samples contained an NRAS mutation. Of note, our results demonstrated the fact that BRAF and NRAS somatic mutations are, largely, mutually exclusive (Fisher’s exact p = 0.0001). Interestingly, 12 of the BRAF^V600^-mutant tumors contained at least one mutation in the PI3K pathway, whereas 6 of the NRAS-mutant tumors contained at least one mutation in the PI3K pathway, demonstrating that MAPK and PI3K pathway mutations frequently co-occur (Figure 3a).

Despite the fact that the BRAF^V600^ mutation was the single most prevalent mutation in our melanoma population (51% of the melanomas had a BRAF^V600^ mutation), 41% of the melanoma tumors contained a PI3K mutation including 10 samples that were wild-type for BRAF^V600^ and NRAS. These PI3K mutations are important to note because they may be a contributing factor to melanoma proliferation and yield valuable clues for personalized treatment options (Figure 3a).

Interestingly, the Sanger-verified somatic mutations demonstrated a high degree of heterogeneity among these PI3K mutations. Sanger sequencing revealed somatic mutations in the known cancer driver genes PIK3CA, and PTEN. Nevertheless, novel somatic mutations were also identified in IRS4, MTOR, NFKB1, PIK3R1, PIK3R4, and PIK3R5 at low frequencies (Figure 3b; Table 2). Of note, major portions of the PI3K pathway mutations were C:G→T:A substitutions (16/40; 40%). This particular mutation signature has previously been reported as a common mutation signature in melanoma [29]. From these results, two major themes about PI3K pathway dysregulation can be demonstrated. First, there are several low frequency mutations in the PI3K pathway that can contribute to unregulated melanocyte proliferation. Second, dysregulation of the PI3K pathway could potentially be caused by mutations in either upstream regulators (PIK3CA, PTEN, IRS4, PIK3R1, PIK3R4, PIK3R5) or downstream effectors (MTOR, NFKB1) of the pathway. The precise location of an activating mutation in an individual tumor may have important implications when targeting the relevant pathways for individualized treatment options.

**Table 2:** Non-synonymous somatic mutations observed by 454FLX and confirmed by Sanger sequencing.

Gene	Chr.	Genomic Position	Nucleotide Alteration	Protein Alteration	Protein Position	Sanger-confirmed Tumors
BRAF	7	143162	G>A	GLY>ARG	469	13T
BRAF	7	171411	A>G	ASP>GLY	594	2T
BRAF	7	171420	T>A	LEU>PHE	597	32T
BRAF	7	171429	T>A	VAL>GLU	600	23T, 43T, 52T, 64T, 26T, 34T, 6T, 5T, 68T, 19T, 4T, 9T, 37T, 49T, 69T, 73T, 80T, 81T, 83T, 84T, 85T, 86T, 88T, 91T, 94T, 98T, 99T, 103T, 105T
BRAF	7	171428–71429	GT>AA	VAL>LYS	600	30T, 16T, 41T, 71T
BRAF	7	171429–171430	TG>AA	VAL>GLU	600	78T, 100T
IRS4	X	3846	C>T	ARG>TRP	1257	7T
IRS4	X	136	C>A	ALA>GLU	20	29T
IRS4	X	3684–3685	GC>AT	ALA>ILE	1203	74T
MTOR	1	149619	C>T	ARG>STOP	2443	13T
MTOR	1	22075	C>T	LEU>PHE	552	44T
NFKB1	4	36559	C>T	PRO>SER	65	52T
NFKB1	4	99601	C>T	HIS>TYR	556	13T
NFKB1	4	105252	C>T	LEU>PHE	611	1T
NFKB1	4	105927	C>T	SER>PHE	685	77T
NRAS	1	2986	C>A	GLN>LYS	61	2T
NRAS	1	2987	A>G	GLN>ARG	61	7T, 12T
NRAS	1	769	G>A	GLY>ASP	12	72T
NRAS	1	771	G>C	GLY>ARG	13	104T
NRAS	1	2986	C>A	GLN>LYS	61	17T, 44T, 63T, 77T
NRAS	1	2987	A>G	GLN>ARG	61	74T, 95T, 24T, 60T
PIK3CA	3	55239	T>G	VAL>GLY	344	4T
PIK3CA	3	85669	G>A	GLU>LYS	1012	52T
PIK3R1	5	67202	G>A	SPLICE SITE	205–206	34T
PIK3R4	3	1954	G>A	ARG>GLN	207	7T
PIK3R4	3	39852	C>T	PRO>SER	890	32T
PIK3R4	3	39859	C>T	SER>PHE	892	1T
PIK3R5	17	24906	G>A	ARG>GLN	563	55T
PIK3R5	17	24975	C>T	SER>PHE	586	24T
PTEN	10	30620	C>T	PRO>SER	38	16T
PTEN	10	62043–62168	DELETION	DELETION	55–70	7T, 18T, 64T & 68T
PTEN	10	94364–94619	DELETION	DELETION	212–267	7T, 18T, 64T & 68T
PTEN	10	97415–97719	DELETION	DELETION	268–342	7T, 18T, 64T & 68T
PTEN	10	101806–102069	DELETION	DELETION	319–320	7T, 18T, 64T & 68T
PTEN	10	97610–97613	ACTT>–	FRAMESHIFT	343	43T
PTEN	10	88813	G>T	GLY>STOP	208	15T
PTEN *	10	67609–67652	DELETION	DELETION	70–84	7T, 18T, 64T
PTEN *	10	69576–69814	DELETION	DELETION	84–164	7T, 64T
PTEN *	10	88681.88822	DELETION	DELETION	164–211	7T, 18T
PTEN *	10	97467	T>C	PHE>SER	812	12T
PTEN*	10	30588–30672	DELETION	DELETION	26–54	22T, 51T, 69T, 88T, 90T, 98T, 103T
PTEN*	10	67609–67652	DELETION	DELETION	70–84	22T, 51T, 88T, 90T, 98T, 103T
PTEN*	10	69580–69814	DELETION	DELETION	84–164	22T, 51T, 69T, 88T, 90T, 98T, 103T
PTEN*	10	88681–88822	DELETION	DELETION	164–211	22T, 51T, 69T, 88T, 90T, 99T
PTEN*	10	94416–94582	DELETION	DELETION	211–267	22T, 51T
PTEN*	10	97457–97681	DELETION	DELETION	267–342	22T, 88T, 90T, 99T

* Discovered by qPCR.

## Discussion

The recent development of mutant BRAF^V600^-targeted chemotherapeutics has offered great opportunities for increased effectiveness in treating metastatic melanoma. However, clinical studies have revealed that resistance is frequently acquired, and many melanomas eventually overcome these agents, leading to clinical progression of disease. In several cell-based and clinical studies, evidence points to the PI3K pathway as an important mechanism driving melanoma survival and proliferation in the presence of BRAF^V600^-targeted therapy [8], [10], [30]. Our melanoma somatic mutation profiling reveals the identities of several MAPK and PI3K pathway components, and how these mutations may interact in individual metastatic melanoma patients.

The single most frequent somatic mutation was BRAF^V600E^. However, it is noteworthy that 17% of the tumors studied contained both a PI3K pathway and a BRAF^V600^ mutation. Functional studies are needed to understand the interactions of mutations in individual tumors and the clinical relevance of inhibiting these two pathways based on the somatic mutations present. Nevertheless, PI3K mutations coupled with BRAF^ V600^ mutations could account for the bypass of the NFA mechanism in the MAPK pathway [20], [21], [22], [23], [24] and, ultimately, the chemotherapeutic resistance of patients treated only with mutant BRAF^V600^ inhibitors.

The individual PI3K pathway mutations discovered in our melanoma DNA samples were also of interest. The most commonly mutated PI3K pathway member was PTEN, with 22% of melanoma patients carrying an inactivating mutation, many of which were homozygous deletions of entire exons. Through these inactivating mutations, PTEN’s negative regulatory activity is lost, enabling the PI3K pathway to induce cell proliferation and counteract apoptosis in an unchecked manner. Recent studies have shown that PTEN loss, coupled with a BRAF^V600^ mutation, can continue cellular progression even when the oncogenic BRAF is inhibited [31]. However, two separate reports have demonstrated that resistance to BRAF^V600^ can be overcome by PI3K pathway inhibition [32], [33].

Nevertheless, there were several novel somatic mutations in the PI3K pathway that are noteworthy. Two of the more intriguing mutated genes were NFKB1 and PIK3R4 (Figure S1). The NFKB1 gene codes for a transcription factor that induces expression of inflammatory cytokines when localized to the nucleus. Studies have shown that this gene is mutated in other cancers, including those of the breast and colon [34], [35]. However, its mechanistic role as a potential cancer driver gene is not well characterized. For the case of PIK3R4, this gene codes for a homolog of the p85á regulatory subunit and has been shown to be involved in endosomal sorting [36].

Overall, in metastatic melanoma, the PI3K pathway is altered by somatic mutations in a wide array of genes including PIK3CA, MTOR, PIK3R1, PIK3R5, and IRS4. PIK3CA and, recently, MTOR have been successfully targeted with selective agents to treat human cancers. Nevertheless, our discovery of novel mutations in recruiter proteins like PIK3R1, PIK3R5, and IRS4 can create additional opportunities for gene-targeted chemotherapeutics.

Because of the several possible mutations involved in melanoma progression, it is clear that targeting BRAF^V600^ mutations alone is likely to be an inadequate strategy for treating melanoma. It will become more important to determine the mutation profile for each patient before deciding on a specific inhibitor-based treatment plan. The benefits of this approach are two-fold. First, if a patient’s mutation status is known, a targeted chemotherapeutic regimen would be much more effective. Second, mutation status could possibly help avoid toxic effects of drugs that not only are ineffective, but also are costly and cause more harm than good to certain patients. Consider a metastatic melanoma patient given a BRAF^V600E^ inhibitor such as PLX-4032 [37]. While our data suggests that the BRAF^V600^ mutations are present in approximately half of metastatic melanoma patients, if mutation status has not been determined and the patient has not only a BRAF^V600^ hotspot mutation, but also a MTOR mutation, disease progression could still result. In fact, when a selective BRAF drug is administered to a patient harboring an NRAS mutation, C-raf, in turn, is activated, driving the cell cycle and promoting disease progression [5]. Such therapy based on incomplete information may actually exacerbate tumor burden and worsen prognosis.

In conclusion, our somatic mutation profiling of the MAPK and PI3K pathway revealed alternative, low frequency mutations in the PI3K pathway that account for melanoma progression, and offer possible explanations for why targeted BRAF^V600^ inhibitors are not completely effective in treating metastatic melanoma patients. Functional characterization of these novel drivers may lead to the development of additional targeted therapies that will improve treatment outcomes for metastatic melanoma patients.

## Supporting Information

Figure S1 **Sample chromatograms of Sanger-verified NFKB1 (a) and PIK3R4 (b) somatic mutations.**
(TIF)

Table S1 **Melanoma patients’ clinical data and gene mutation profiles.**
(DOCX)

Table S2 **454FLX variants detected in melanoma pools.** Discovery, prevalence 1, and prevalence 2 pools of melanoma genomic DNA were subjected to gene mutation profiling. Sequence reads were aligned to HG19/B37 reference sequence. Variants greater than 1% minor allele frequency are reported. Variants found in dbSNP, HapMap, or the 1000 Genomes database were annotated as germline. All other variants were annotated as Somatic.
(DOCX)
