# Supplementary figures and images for: Novel Somatic Mutations to PI3K Pathway Genes in Metastatic Melanoma
Source: PLoS One. 2012 Aug 17;7(8):e43369. doi: 10.1371/journal.pone.0043369 (PMC3422312; doi:10.1371/journal.pone.0043369)

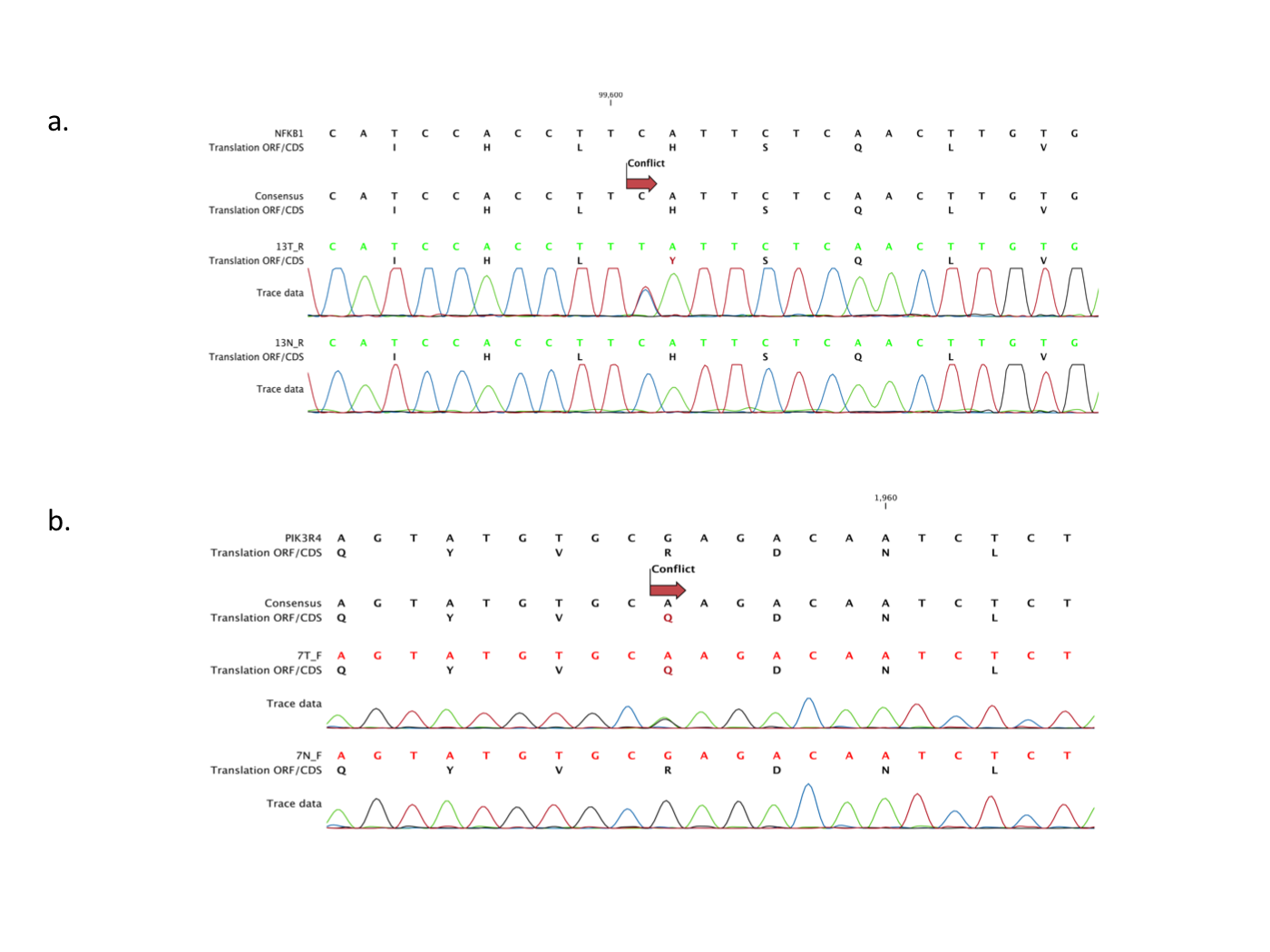

Supplement: Figure S1 — Sample chromatograms of Sanger-verified NFKB1 (a) and PIK3R4 (b) somatic mutations. (TIF) [file pone.0043369.s001.tif]
