# Supplementary material for: Novel Somatic Mutations to PI3K Pathway Genes in Metastatic Melanoma
Source: PLoS One. 2012 Aug 17;7(8):e43369. doi: 10.1371/journal.pone.0043369 (PMC3422312; doi:10.1371/journal.pone.0043369)
Supplement: Table S1 — Melanoma patients’ clinical data and gene mutation profiles. (DOCX) [file pone.0043369.s002.docx]

| **Tumor Identifier** | **Site of Primary** | **Primary Tumor Site** | **Metastatic Tumor Site** | **Characteristic** | **Patient Gender** | **Patient Age (years)** | **BRAF** | **NRAS** | **PIK3CA** | **IRS4** | **PIK3R1** | **PIK3R4** | **PIK3R5** | **PTEN** | **MTOR** | **NFKB1** |
| --- | --- | --- | --- | --- | --- | --- | --- | --- | --- | --- | --- | --- | --- | --- | --- | --- |
| 1T | Skin of scalp and neck | Posterior neck | Lung | cutaneous | F | 29 |  |  |  |  |  | S892F |  |  |  | L611F |
| 2T | Skin of trunk | Upper back | Pectoral, subcutaneous | cutaneous | M | 30 | N594G | Q61K |  |  |  |  |  |  |  |  |
| 3T | Skin of scalp and neck | Scalp | Forehead, subcutaneous | cutaneous | M | 18 |  |  |  |  |  |  |  |  |  |  |
| 4T | Skin of arm and shoulder | Arm | Supraclavicular, soft tissue | cutaneous | F | 33 | V600E |  | V344G |  |  |  |  |  |  |  |
| 5T | Skin of trunk | Lower abdomen | Iliac | cutaneous | M | 47 | V600E |  |  |  |  |  |  |  |  |  |
| 6T | Skin, other and unspecified parts of face | Temple | Neck, soft tissue | cutaneous | M | 42 | V600E |  |  |  |  |  |  |  |  |  |
| 7T | Skin of leg and hip | Knee | Stomach | cutaneous | M | 53 |  | Q61R |  | R1257W |  | R207Q |  | DEL |  |  |
| 9T | Skin of trunk | Mid Back | Back, subcutaneous | cutaneous | M | 62 | V600E |  |  |  |  |  |  |  |  |  |
| 10T | Skin of trunk | Back | Axilla | cutaneous | M | 55 |  |  |  |  |  |  |  |  |  |  |
| 12T | Skin of arm and shoulder | Arm | Upper arm, subcutaneous | cutaneous | M | 53 |  | Q61R |  |  |  |  |  | F812S |  |  |
| 13T | Skin, other and unspecified parts of face | Forehead | Chest wall, subcutaneous | cutaneous | M | 49 | G469R |  |  |  |  |  |  |  | R2443* | H556Y |
| 14T | Skin of leg and hip | Foot | Small Bowel | cutaneous | F | 58 |  |  |  |  |  |  |  |  |  |  |
| 15T | Skin of leg and hip | Heel | Thigh, subcutaneous | cutaneous | M | 39 |  |  |  |  |  |  |  | G208* |  |  |
| 16T | Skin of scalp and neck | Scalp | Lung | cutaneous | M | 62 | V600K |  |  |  |  |  |  | DEL |  |  |
| 17T | Skin, NOS | Unknown | Shoulder, subcutaneous | cutaneous | M | 33 |  | Q61K |  |  |  |  |  |  |  |  |
| 18T | Skin of trunk | Upper back | Clavicle, soft tissue | cutaneous | M | 55 |  |  |  |  |  |  |  | DEL |  |  |
| 19T | Skin of arm and shoulder | Wrist | Scapula, subcutaneous | cutaneous | M | 49 | V600E |  |  |  |  |  |  |  |  |  |
| 22T | Skin of trunk | Nipple | Chest wall, subcutaneous | cutaneous | M | 51 |  |  |  |  |  |  |  | DEL |  |  |
| 23T | Skin of scalp and neck | Scalp | Lung | cutaneous | M | 44 | V600E |  |  |  |  |  |  |  |  |  |
| 24T | Skin of arm and shoulder | Upper arm | Axilla | cutaneous | M | 49 |  | Q61R |  |  |  |  | S586F |  |  |  |
| 26T | Skin of leg and hip | Upper thigh | Lung | cutaneous | F | 48 | V600E |  |  |  |  |  |  |  |  |  |
| 29T | Skin of leg and hip | Thigh | Inguinal | cutaneous | M | 51 |  |  |  | A20E |  |  |  |  |  |  |
| 30T | Skin of trunk | Upper back | Lung | cutaneous | F | 53 | V600K |  |  |  |  |  |  |  |  |  |
| 31T | Skin of leg and hip | Knee | Thigh, subcutaneous | cutaneous | F | 49 |  |  |  |  |  |  |  |  |  |  |
| 32T | Skin of arm and shoulder | Shoulder | Omentum | cutaneous | M | 58 | L597F |  |  |  |  | P890S |  |  |  |  |
| 34T | Skin of trunk | Mid back | Shoulder, subcutaneous | cutaneous | M | 31 | V600E |  |  |  | SPLICE SITE |  |  |  |  |  |
| 35T | Skin of leg and hip | Heel | Thigh, subcutaneous | cutaneous | F | 23 |  |  |  |  |  |  |  |  |  |  |
| 36T | Skin of arm and shoulder | Shoulder | Thigh, subcutaneous | cutaneous | M | 25 |  |  |  |  |  |  |  |  |  |  |
| 37T | Skin of scalp and neck | Neck | Omentum | cutaneous | F | 38 | V600E |  |  |  |  |  |  |  |  |  |
| 41T | Skin of scalp and neck | Scalp | Neck, soft tissue | cutaneous | M | 45 | V600K |  |  |  |  |  |  |  |  |  |
| 43T | Skin of leg and hip | Thigh | Popliteal soft tissue | cutaneous | F | 19 | V600E |  |  |  |  |  |  | FRAME  SHIFT |  |  |
| 44T | Skin of trunk | Mid back | Lung | cutaneous | M | 56 |  | Q61K |  |  |  |  |  |  | L552F |  |
| 45T | Skin of trunk | Peri-umbilicus | Mediastinum | cutaneous | M | 48 |  |  |  |  |  |  |  |  |  |  |
| 49T | Skin of trunk | Lower back | Thigh, subcutaneous | cutaneous | M | 43 | V600E |  |  |  |  |  |  |  |  |  |
| 50T | Skin, NOS | Unknown | Inguinal | cutaneous | F | 49 |  |  |  |  |  |  |  |  |  |  |
| 51T | Skin of leg and hip | Medial thigh | Adnexa | cutaneous | F | 50 |  |  |  |  |  |  |  | DEL |  |  |
| 52T | Skin of arm and shoulder | Shoulder | Lung | cutaneous | F | 39 | V600E |  | E1012K |  |  |  |  |  |  | P65S |
| 55T | Skin of scalp and neck | Neck | Lung | cutaneous | M | 60 |  |  |  |  |  |  | R563Q |  |  |  |
| 60T | Skin of trunk | Abdomen | Flank, subcutaneous | cutaneous | M | 46 |  | Q61R |  |  |  |  |  |  |  |  |
| 63T | Skin, other and unspecified parts of face | Mandible | Small Bowel | cutaneous | M | 30 |  | Q61K |  |  |  |  |  |  |  |  |
| 64T | Skin, NOS | Unknown | Ovary | cutaneous | F | 32 | V600E |  |  |  |  |  |  | DEL |  |  |
| 68T | Skin of leg and hip | Knee | Lung | cutaneous | M | 49 | V600E |  |  |  |  |  |  | DEL |  |  |
| 69T | Skin of leg and hip | Thigh | Axilla | cutaneous | M | 36 | V600E |  |  |  |  |  |  | DEL |  |  |
| 71T | Skin of arm and shoulder | Anterior shoulder | Lung | cutaneous | M | 67 | V600K |  |  |  |  |  |  |  |  |  |
| 72T | Skin of trunk | Mid back | Liver | cutaneous | M | 53 |  | G12N |  |  |  |  |  |  |  |  |
| 73T | Skin of trunk | Scapula | Breast | cutaneous | F | 45 | V600E |  |  |  |  |  |  |  |  |  |
| 74T | Skin of leg and hip | Leg | Lower extremity, subcutaneous | cutaneous | F | 40 |  | Q61R |  | A1203I |  |  |  |  |  |  |
| 77T | Skin of trunk | Posterior shoulder | Lung | cutaneous | M | 39 |  | Q61K |  |  |  |  |  |  |  | S685F |
| 78T | Skin of trunk | Back | Lung | cutaneous | F | 27 | V600E |  |  |  |  |  |  |  |  |  |
| 80T | Skin of leg and hip | Calf | Popliteal | cutaneous | M | 36 | V600E |  |  |  |  |  |  |  |  |  |
| 81T | Skin of arm and shoulder | Arm | Upper arm, subcutaneous | cutaneous | F | 60 | V600E |  |  |  |  |  |  |  |  |  |
| 83T | Skin of arm and shoulder | Arm | Back, subcutaneous | cutaneous | F | 33 | V600E |  |  |  |  |  |  |  |  |  |
| 84T | Skin of leg and hip | Calf | Thigh, subcutaneous | cutaneous | F | 60 | V600E |  |  |  |  |  |  |  |  |  |
| 85T | Skin of trunk | Anterior chest wall | Chest wall, subcutaneous | cutaneous | M | 44 | V600E |  |  |  |  |  |  |  |  |  |
| 86T | Skin of arm and shoulder | Forearm | Liver | cutaneous | F | 42 | V600E |  |  |  |  |  |  |  |  |  |
| 88T | Skin of scalp and neck | Scalp | Chest wall, subcutaneous | cutaneous | F | 37 | V600E |  |  |  |  |  |  | DEL |  |  |
| 90T | Skin of scalp and neck | Occipital scalp | Neck, soft tissue | cutaneous | M | 19 |  |  |  |  |  |  |  | DEL |  |  |
| 91T | Skin of arm and shoulder | Shoulder | Subcostal soft tissue | cutaneous | F | 55 | V600E |  |  |  |  |  |  |  |  |  |
| 94T | Skin, NOS | Unknown | Adrenal gland | cutaneous | M | 44 | V600E |  |  |  |  |  |  |  |  |  |
| 95T | Skin, NOS | Unknown | Inguinal | cutaneous | F | 58 |  | Q61R |  |  |  |  |  |  |  |  |
| 96T | Skin, NOS | Unknown | Inguinal | cutaneous | M | 49 |  |  |  |  |  |  |  |  |  |  |
| 98T | Skin of leg and hip | Foot | Small Bowel | cutaneous | F | 58 | V600E |  |  |  |  |  |  | DEL |  |  |
| 99T | Skin of trunk | Back | Liver | cutaneous | M | 57 | V600E |  |  |  |  |  |  | DEL |  |  |
| 100T | Skin of trunk | Back | Chest wall, soft tissue | cutaneous | M | 28 | V600E |  |  |  |  |  |  |  |  |  |
| 103T | Skin of arm and shoulder | Shoulder | Axilla | cutaneous | F | 35 | V600E |  |  |  |  |  |  | DEL |  |  |
| 104T | Skin of leg and hip | Ankle | Thigh, subcutaneous | cutaneous | M | 56 |  | G13R |  |  |  |  |  |  |  |  |
| 105T | Skin of trunk | Upper back | Neck, soft tissue | cutaneous | M | 28 | V600E |  |  |  |  |  |  |  |  |  |
