# Supplementary material for: Novel Somatic Mutations to PI3K Pathway Genes in Metastatic Melanoma
Source: PLoS One. 2012 Aug 17;7(8):e43369. doi: 10.1371/journal.pone.0043369 (PMC3422312; doi:10.1371/journal.pone.0043369)
Supplement: Table S2 — 454FLX variants detected in melanoma pools. Discovery, prevalence 1, and prevalence 2 pools of melanoma genomic DNA were subjected to gene mutation profiling. Sequence reads were aligned to HG19/B37 reference sequence. Variants greater than 1% minor allele frequency are reported. Variants found in dbSNP, HapMap, or the 1000 Genomes database were annotated as germline. All other variants were annotated as Somatic. (DOCX) [file pone.0043369.s003.docx]

| Pool | Chr | Region | Ref. | Allele Variation | major allele frequency | minor allele frequency | major allele count | minor allele count | Amino Acid Change | Non-synonymous | CDS | Germline | Somatic |
| --- | --- | --- | --- | --- | --- | --- | --- | --- | --- | --- | --- | --- | --- |
| Discovery | 1 | 11172948 | G | G/A | 98.9 | 1.1 | 5860 | 67 | p.[=];[Arg2443*] | Yes | MTOR |  | X |
| Discovery | 1 | 11181327 | C | C/T | 78.9 | 21.1 | 1644 | 440 |  | No | MTOR | X |  |
| Discovery | 1 | 11190646 | G | G/A | 85.6 | 14.4 | 798 | 134 |  | No | MTOR | X |  |
| Discovery | 1 | 11205058 | C | T/C | 78.1 | 21.9 | 2166 | 608 |  | No | MTOR | X |  |
| Discovery | 1 | 11288758 | G | A/G | 79 | 20.9 | 590 | 156 |  | No | MTOR | X |  |
| Discovery | 1 | 11301714 | A | G/A | 78.1 | 21.9 | 1300 | 364 |  | No | MTOR | X |  |
| Discovery | 1 | 11307713 | G | G/A | 98.1 | 1.9 | 2324 | 45 |  | No | MTOR |  | X |
| Discovery | 1 | 115256529 | T | T/C | 92.9 | 7.1 | 4109 | 315 | p.[=];[Gln61Arg] | Yes | NRAS |  | X |
| Discovery | 1 | 115256530 | G | G/T | 98.6 | 1.4 | 4345 | 61 | p.[=];[Gln61Lys] | Yes | NRAS |  | X |
| Discovery | 2 | 233990594 | A | A/G | 98.7 | 1.3 | 743 | 10 |  | No | SHIP1 | X |  |
| Discovery | 2 | 234079711 | G | G/A | 98.6 | 1.4 | 1050 | 15 | p.[=, =];[Gly388Arg, Gly388Arg] | Yes | SHIP1 |  | X |
| Discovery | 2 | 234079712 | G | G/A | 98.6 | 1.4 | 1051 | 15 | p.[=, =];[Gly388Glu, Gly388Glu] | Yes | SHIP1 |  | X |
| Discovery | 2 | 234093058 | C | C/T | 99 | 1 | 3383 | 33 | p.[=, =];[Arg476Trp, Arg476Trp] | Yes | SHIP1 |  | X |
| Discovery | 2 | 234104102 | C | C/A | 94.2 | 5.7 | 2475 | 150 | p.[=, =];[His638Gln, His638Gln] | Yes | SHIP1 |  | X |
| Discovery | 2 | 234112976 | C | C/T | 98.7 | 1.2 | 2468 | 31 | p.[=, =];[Arg814Cys, Arg814Cys] | Yes | SHIP1 |  | X |
| Discovery | 2 | 234113057 | G | G/A | 94.5 | 5.5 | 1089 | 63 | p.[=, =];[Val841Met, Val841Met] | Yes | SHIP1 | X |  |
| Discovery | 3 | 130449222 | A | A/G | 85.9 | 14.1 | 1170 | 192 |  | No | PIK3R4 | X |  |
| Discovery | 3 | 130454828 | A | A/G | 98.9 | 1.1 | 4523 | 51 | p.[=];[Leu251Pro] | Yes | PIK3R4 |  | X |
| Discovery | 3 | 130463743 | C | C/T | 98.9 | 1.1 | 1601 | 17 | p.[=];[Arg107Gln] | Yes | PIK3R4 |  | X |
| Discovery | 3 | 178916894 | T | T/C | 97.6 | 2.4 | 1003 | 25 | p.[=];[Leu94Pro] | Yes | PIK3CA |  | X |
| Discovery | 3 | 178921549 | T | T/G | 97.3 | 2.7 | 2710 | 74 | p.[=];[Val344Gly] | Yes | PIK3CA |  | X |
| Discovery | 3 | 178927410 | A | A/G | 95 | 5 | 1640 | 86 | p.[=];[Ile391Met] | Yes | PIK3CA | X |  |
| Discovery | 4 | 55593464 | A | A/C | 93 | 7 | 3610 | 273 | p.[=, =];[Met537Leu, Met541Leu] | Yes | KIT | X |  |
| Discovery | 4 | 55593481 | A | A/G | 94.2 | 5.8 | 3846 | 236 |  | No | KIT | X |  |
| Discovery | 4 | 55599268 | C | C/T | 96.7 | 3.3 | 625 | 21 |  | No | KIT | X |  |
| Discovery | 4 | 55602765 | G | G/C | 91.8 | 8.2 | 1375 | 123 |  | No | KIT | X |  |
| Discovery | 4 | 55604597 | T | T/A | 98.3 | 1.7 | 893 | 15 |  | No | KIT |  | X |
| Discovery | 4 | 103504087 | T | T/A | 98.5 | 1.5 | 3183 | 48 | p.[=, =];[Asp300Glu, Asp299Glu] | Yes | NFKB1 |  | X |
| Discovery | 4 | 103504088 | T | T/A | 99 | 1 | 3198 | 32 | p.[=, =];[Phe301Ile, Phe300Ile] | Yes | NFKB1 |  | X |
| Discovery | 4 | 103505961 | C | C/T | 97 | 3 | 2472 | 77 |  | No | NFKB1 | X |  |
| Discovery | 4 | 103514658 | T | C/T | 94.9 | 5.1 | 1781 | 96 |  | No | NFKB1 | X |  |
| Discovery | 4 | 103518700 | A | A/G | 98.2 | 1.8 | 3528 | 63 | p.[=, =];[Met507Val, Met506Val] | Yes | NFKB1 | X |  |
| Discovery | 4 | 153268105 | G | G/A | 98.3 | 1.7 | 1015 | 18 | p.[=, =, =];[Gln155*, Gln117*, Gln235*] | Yes | FBXW7 |  | X |
| Discovery | 5 | 67575548 | T | T/C | 98.7 | 1.3 | 1486 | 19 |  | No | PIK3R1 | X |  |
| Discovery | 5 | 67588148 | G | G/A | 97.8 | 2.2 | 863 | 19 | p.[=, =, =];[Met326Ile, Met26Ile, Met56Ile] | Yes | PIK3R1 | X |  |
| Discovery | 5 | 67589197 | A | A/G | 98.7 | 1.3 | 1341 | 18 |  | No | PIK3R1 |  | X |
| Discovery | 5 | 67592104 | G | G/A | 96.7 | 3.3 | 1185 | 41 |  | No | PIK3R1 |  | X |
| Discovery | 6 | 166826255 | G | A/G | 64.7 | 35.3 | 22 | 12 |  | No | RPS6KA2 | X |  |
| Discovery | 6 | 166826304 | T | C | 76.5 |  | 26 |  |  | No | RPS6KA2 | X |  |
| Discovery | 6 | 166827378 | G | G/A | 97.8 | 2.2 | 1633 | 37 |  | No | RPS6KA2 |  | X |
| Discovery | 6 | 166862233 | T | T/C | 58.8 | 41.2 | 1028 | 719 |  | No | RPS6KA2 | X |  |
| Discovery | 6 | 166873010 | C | C/T | 88.3 | 11.7 | 1282 | 170 |  | No | RPS6KA2 | X |  |
| Discovery | 6 | 166904212 | C | C/T | 91.3 | 8.7 | 356 | 34 |  | No | RPS6KA2 | X |  |
| Discovery | 6 | 166912062 | C | C/T | 92.4 | 7.6 | 635 | 52 |  | No | RPS6KA2 | X |  |
| Discovery | 6 | 166952264 | G | A/G | 90.2 | 9.8 | 1776 | 192 |  | No | RPS6KA2 | X |  |
| Discovery | 6 | 167271711 | T | C/T | 97.8 | 2.2 | 862 | 19 | p.[Thr34Ala];[=] | Yes | RPS6KA2 | X |  |
| Discovery | 6 | 167271716 | T | C/T | 75.5 | 24.5 | 664 | 215 | p.[Glu32Gly];[=] | Yes | RPS6KA2 | X |  |
| Discovery | 7 | 55214348 | C | C/T | 54.9 | 45.1 | 100 | 82 |  | No | EGFR | X |  |
| Discovery | 7 | 55219021 | C | C/T | 97.5 | 2.5 | 789 | 20 |  | No | EGFR |  | X |
| Discovery | 7 | 55229255 | G | G/A | 75.4 | 24.6 | 1937 | 631 | p.[=, =, =];[Arg521Lys, Arg521Lys, Arg521Lys] | Yes | EGFR | X |  |
| Discovery | 7 | 55233089 | C | C/T | 97.4 | 2.6 | 413 | 11 |  | No | EGFR | X |  |
| Discovery | 7 | 55238874 | T | T/A | 60.7 | 39.3 | 429 | 278 |  | No | EGFR | X |  |
| Discovery | 7 | 55249063 | G | A/G | 62.6 | 37.4 | 196 | 117 |  | No | EGFR | X |  |
| Discovery | 7 | 55259450 | C | C/T | 96.4 | 3.6 | 352 | 13 |  | No | EGFR | X |  |
| Discovery | 7 | 55266417 | T | C/T | 95.5 | 4.5 | 571 | 27 |  | No | EGFR | X |  |
| Discovery | 7 | 55268916 | C | C/T | 86.1 | 13.9 | 576 | 93 |  | No | EGFR | X |  |
| Discovery | 7 | 140453136 | A | A/T | 60.4 | 39.6 | 2307 | 1511 | p.[=];[Val600Glu] | Yes | BRAF |  | X |
| Discovery | 7 | 140453137 | C | C/T | 91.8 | 8.2 | 3925 | 350 | p.[=];[Val600Met] | Yes | BRAF |  | X |
| Discovery | 7 | 140481403 | C | C/T | 98.4 | 1.6 | 5347 | 89 | p.[=];[Gly469Arg] | Yes | BRAF |  | X |
| Discovery | 7 | 140534494 | T | T/C | 98.9 | 1.1 | 4780 | 54 | p.[=];[Asn140Ser] | Yes | BRAF |  | X |
| Discovery | 9 | 131893861 | G | G/C | 97.6 | 2.4 | 2455 | 60 | p.[=, =, =, =, =];[Val93Leu, Val135Leu, Val135Leu, Val170Leu, Val100Leu] | Yes | PP2A |  | X |
| Discovery | 9 | 131909736 | C | C/T | 91.1 | 8.9 | 1171 | 115 | p.[=, =, =, =, =];[Ser280Leu, Ser322Leu, Ser322Leu, Ser357Leu, Ser287Leu] | Yes | PP2A | X |  |
| Discovery | 9 | 135772717 | G | G/A | 96.1 | 3.9 | 247 | 10 |  | No | TSC1 | X |  |
| Discovery | 9 | 135779052 | G | G/A | 93.9 | 6.1 | 526 | 34 | p.[=, =, =];[His681Tyr, His731Tyr, His732Tyr] | Yes | TSC1 | X |  |
| Discovery | 9 | 135782221 | T | T/C | 77.9 | 22.1 | 575 | 163 |  | No | TSC1 | X |  |
| Discovery | 9 | 135786904 | A | A/G | 84.1 | 15.9 | 1228 | 232 | p.[=, =, =];[Met271Thr, Met322Thr, Met322Thr] | Yes | TSC1 | X |  |
| Discovery | 9 | 135797338 | G | G/A | 98.6 | 1.4 | 2702 | 39 |  | No | TSC1 |  | X |
| Discovery | 10 | 89653814 | C | C/T | 93.7 | 6.3 | 582 | 39 | p.[=];[Pro38Ser] | Yes | PTEN |  | X |
| Discovery | 10 | 101977883 | C | T/C | 77.6 | 22.4 | 280 | 81 | p.[Val268Ile];[=] | Yes | CHUK | X |  |
| Discovery | 12 | 5721919 | C | A | 100 |  | 10 |  | p.Ala664Ser | Yes | ANO2 |  | X |
| Discovery | 13 | 41134350 | A | A/C | 98.3 | 1.7 | 1385 | 24 | p.[=];[Tyr426*] | Yes | FOXO1 |  | X |
| Discovery | 13 | 41134356 | A | A/C | 98.3 | 1.7 | 1389 | 24 | p.[=];[Tyr424*] | Yes | FOXO1 |  | X |
| Discovery | 13 | 41134358 | A | A/G | 98.4 | 1.6 | 1385 | 23 | p.[=];[Tyr424His] | Yes | FOXO1 |  | X |
| Discovery | 13 | 41134364 | G | G/T | 97.3 | 2.7 | 1320 | 36 | p.[=];[Gln422Lys] | Yes | FOXO1 |  | X |
| Discovery | 13 | 41134366 | T | T/C | 97.2 | 2.8 | 1302 | 38 | p.[=];[Tyr421Cys] | Yes | FOXO1 |  | X |
| Discovery | 13 | 41134380 | T | T/C | 97.3 | 2.7 | 1385 | 38 |  | No | FOXO1 |  | X |
| Discovery | 13 | 41134388 | A | A/G | 97.2 | 2.8 | 1379 | 39 |  | No | FOXO1 |  | X |
| Discovery | 13 | 41134405 | G | G/A | 96.3 | 3.7 | 1381 | 53 | p.[=];[Ala408Val] | Yes | FOXO1 |  | X |
| Discovery | 13 | 41134410 | C | C/G | 96.4 | 3.6 | 1378 | 52 |  | No | FOXO1 |  | X |
| Discovery | 13 | 41134419 | C | C/T | 96.1 | 3.9 | 1379 | 56 |  | No | FOXO1 |  | X |
| Discovery | 13 | 41134458 | C | C/G | 98.6 | 1.4 | 721 | 10 |  | No | FOXO1 |  | X |
| Discovery | 13 | 110436861 | A | T/A | 59.5 | 40.5 | 25 | 17 | p.[Cys514Ser];[=] | Yes | IRS2 | X |  |
| Discovery | 13 | 110436864 | A | T/A | 58.1 | 41.9 | 25 | 18 | p.[Phe513Ile];[=] | Yes | IRS2 | X |  |
| Discovery | 13 | 110437107 | A | T/A | 63.6 | 36.4 | 35 | 20 | p.[Ser432Thr];[=] | Yes | IRS2 |  | X |
| Discovery | 14 | 105239894 | C | C/T | 89.8 | 10.2 | 1360 | 154 |  | No | AKT1 | X |  |
| Discovery | 16 | 2106725 | C | C/G | 98.7 | 1.3 | 1724 | 22 |  | No | TSC2 | X |  |
| Discovery | 16 | 2110805 | G | G/A | 98.7 | 1.3 | 1788 | 24 |  | No | TSC2 | X |  |
| Discovery | 16 | 2114407 | C | C/T | 92 | 8 | 493 | 43 |  | No | TSC2 | X |  |
| Discovery | 16 | 2121869 | C | C/T | 96.6 | 3.4 | 1014 | 36 |  | No | TSC2 | X |  |
| Discovery | 16 | 2121911 | C | C/T | 98.9 | 1.1 | 1102 | 12 |  | No | TSC2 |  | X |
| Discovery | 16 | 2125834 | T | T/C | 98.4 | 1.6 | 3049 | 50 |  | No | TSC2 | X |  |
| Discovery | 16 | 2126529 | C | C/A | 98.2 | 1.8 | 588 | 11 | p.[=, =, =];[Thr927Asn, Thr927Asn, Thr927Asn] | Yes | TSC2 |  | X |
| Discovery | 16 | 2138269 | T | T/C | 79.4 | 20.6 | 1834 | 475 |  | No | TSC2 | X |  |
| Discovery | 16 | 2138584 | G | G/C | 85.7 | 14.3 | 359 | 60 |  | No | TSC2 | X |  |
| Discovery | 17 | 8790849 | C | C/T | 97.8 | 2.2 | 1663 | 37 | p.[=, =];[Ala590Thr, Ala590Thr] | Yes | PIK3R5 |  | X |
| Discovery | 17 | 8790929 | C | C/T | 98.8 | 1.2 | 1693 | 20 | p.[=, =];[Arg563Gln, Arg563Gln] | Yes | PIK3R5 |  | X |
| Discovery | 17 | 8792514 | G | G/A | 53.1 | 46.9 | 839 | 741 |  | No | PIK3R5 | X |  |
| Discovery | 17 | 8809025 | G | G/A | 98.3 | 1.7 | 2128 | 36 |  | No | PIK3R5 | X |  |
| Discovery | 17 | 8814719 | G | G/A | 98 | 2 | 1074 | 22 |  | No | PIK3R5 | X |  |
| Discovery | 17 | 58011536 | G | G/A | 98.9 | 1.1 | 1659 | 18 | p.[=];[Val232Met] | Yes | RPS6KB1 |  | X |
| Discovery | 17 | 58011843 | G | G/A | 99 | 1 | 2420 | 25 | p.[=];[Trp276*] | Yes | RPS6KB1 | X |  |
| Discovery | 17 | 58022775 | A | A/T | 97.9 | 2.1 | 1427 | 31 |  | No | RPS6KB1 |  | X |
| Discovery | 22 | 22162126 | A | A/G | 90.9 | 9.1 | 996 | 100 |  | No | MAPK1 | X |  |
| Discovery | X | 70321215 | G | G/A | 99 | 1 | 1328 | 14 | p.[=, =];[Ala379Thr, Ala324Thr] | Yes | FOXO4 |  | X |
| Discovery | X | 107975806 | G | G/A | 94.9 | 5.1 | 888 | 48 | p.[=];[Arg1257Trp] | Yes | IRS4 | X |  |
| Discovery | X | 107976940 | G | G/C | 86.3 | 13.7 | 226 | 36 | p.[=];[His879Asp] | Yes | IRS4 | X |  |
| Discovery | X | 107979437 | G | G/A | 97.8 | 2.2 | 655 | 15 |  | No | IRS4 | X |  |
| Discovery | X | 107979475 | G | G/A | 98.2 | 1.8 | 656 | 12 | p.[=];[Leu34Phe] | Yes | IRS4 | X |  |
| Discovery | X | 107979512 | C | C/T | 70.6 | 29.4 | 468 | 195 |  | No | IRS4 | X |  |
| Discovery | X | 107979515 | C | C/T | 72 | 28 | 478 | 186 |  | No | IRS4 | X |  |
| Prevalence One | 1 | 11181327 | C | C/T | 76.1 | 23.9 | 1142 | 359 |  | No | MTOR | X |  |
| Prevalence One | 1 | 11182158 | A | A/C | 98.6 | 1.4 | 2360 | 34 | p.[=];[Leu2230Val] | Yes | MTOR |  | X |
| Prevalence One | 1 | 11190646 | G | G/A | 75.9 | 24.1 | 432 | 137 |  | No | MTOR | X |  |
| Prevalence One | 1 | 11204786 | G | G/A | 97.6 | 2.4 | 2525 | 62 |  | No | MTOR |  | X |
| Prevalence One | 1 | 11205058 | C | T/C | 77.4 | 22.6 | 2262 | 660 |  | No | MTOR | X |  |
| Prevalence One | 1 | 11288758 | G | A/G | 77.2 | 22.6 | 400 | 117 |  | No | MTOR | X |  |
| Prevalence One | 1 | 11300492 | G | G/A | 97.2 | 2.8 | 1484 | 43 | p.[=];[Leu552Phe] | Yes | MTOR |  | X |
| Prevalence One | 1 | 11301714 | A | G/A | 76.9 | 23.1 | 2222 | 666 |  | No | MTOR | X |  |
| Prevalence One | 1 | 115256529 | T | T/C | 86.9 | 13 | 748 | 112 | p.[=];[Gln61Arg] | Yes | NRAS |  | X |
| Prevalence One | 1 | 115256530 | G | G/T | 95.5 | 4.5 | 820 | 39 | p.[=];[Gln61Lys] | Yes | NRAS |  | X |
| Prevalence One | 1 | 115258747 | C | C/T | 97.9 | 2.1 | 1702 | 37 | p.[=];[Gly12Asp] | Yes | NRAS |  | X |
| Prevalence One | 2 | 233990594 | A | A/G | 95.6 | 4.4 | 216 | 10 |  | No | SHIP1 | X |  |
| Prevalence One | 2 | 234113057 | G | G/A | 79.4 | 20.6 | 679 | 176 | p.[=, =];[Val841Met, Val841Met] | Yes | SHIP1 | X |  |
| Prevalence One | 2 | 234113301 | C | C/T | 75.6 | 24.4 | 510 | 165 | p.[=, =];[Ala922Val, Ala922Val] | Yes | SHIP1 | X |  |
| Prevalence One | 3 | 130425838 | G | G/A | 96.2 | 3.8 | 871 | 34 | p.[=];[Ser892Phe] | Yes | PIK3R4 |  | X |
| Prevalence One | 3 | 130425845 | G | G/A | 98.7 | 1.3 | 895 | 12 | p.[=];[Pro890Ser] | Yes | PIK3R4 |  | X |
| Prevalence One | 3 | 130449222 | A | A/G | 88.6 | 11.4 | 643 | 83 |  | No | PIK3R4 | X |  |
| Prevalence One | 3 | 178916898 | T | T/C | 91 | 9 | 483 | 48 |  | No | PIK3CA |  | X |
| Prevalence One | 3 | 178916899 | C | C/T | 94.6 | 5.4 | 609 | 35 | p.[=];[Gln96*] | Yes | PIK3CA |  | X |
| Prevalence One | 3 | 178927410 | A | A/G | 93.2 | 6.8 | 1116 | 82 | p.[=];[Ile391Met] | Yes | PIK3CA | X |  |
| Prevalence One | 3 | 178936092 | A | A/C | 98.7 | 1.1 | 866 | 10 | p.[=];[Glu545Ala] | Yes | PIK3CA |  | X |
| Prevalence One | 3 | 178951998 | A | A/G | 97.6 | 2.4 | 2109 | 52 | p.[=];[Asp1018Gly] | Yes | PIK3CA |  | X |
| Prevalence One | 4 | 55593464 | A | A/C | 89.3 | 10.7 | 1490 | 178 | p.[=, =];[Met537Leu, Met541Leu] | Yes | KIT | X |  |
| Prevalence One | 4 | 55599268 | C | C/T | 94.1 | 5.9 | 1184 | 74 |  | No | KIT | X |  |
| Prevalence One | 4 | 55602765 | G | G/C | 86.5 | 13.5 | 1518 | 236 |  | No | KIT | X |  |
| Prevalence One | 4 | 55604597 | T | T/A | 96.7 | 3.3 | 952 | 32 |  | No | KIT |  | X |
| Prevalence One | 4 | 103451019 | T | T/C | 88.3 | 11.7 | 2116 | 281 |  | No | NFKB1 |  | X |
| Prevalence One | 4 | 103505961 | C | C/T | 92.1 | 7.9 | 915 | 79 |  | No | NFKB1 | X |  |
| Prevalence One | 4 | 103514658 | T | C/T | 94.9 | 5.1 | 1166 | 63 |  | No | NFKB1 | X |  |
| Prevalence One | 4 | 103527731 | C | C/T | 96.4 | 3.6 | 1001 | 37 | p.[=, =];[Leu611Phe, Leu610Phe] | Yes | NFKB1 |  | X |
| Prevalence One | 5 | 67522722 | C | C/T | 94.5 | 5.5 | 207 | 12 |  | No | PIK3R1 | X |  |
| Prevalence One | 5 | 67575548 | T | T/C | 92.2 | 7.8 | 674 | 57 |  | No | PIK3R1 | X |  |
| Prevalence One | 5 | 67588148 | G | G/A | 88.4 | 11.6 | 829 | 109 | p.[=, =, =];[Met326Ile, Met26Ile, Met56Ile] | Yes | PIK3R1 | X |  |
| Prevalence One | 7 | 140453136 | A | A/T | 56.7 | 43.3 | 1389 | 1062 | p.[=];[Val600Glu] | Yes | BRAF |  | X |
| Prevalence One | 7 | 140453137 | C | C/T | 97.4 | 2.6 | 2501 | 68 | p.[=];[Val600Met] | Yes | BRAF |  | X |
| Prevalence One | 7 | 140453145 | A | A/T | 94 | 5.9 | 2403 | 152 | p.[=];[Leu597Gln] | Yes | BRAF |  | X |
| Prevalence One | 7 | 140481412 | C | C/T | 97.2 | 2.8 | 1858 | 53 | p.[=];[Gly466Arg] | Yes | BRAF |  | X |
| Prevalence One | 7 | 140494148 | G | G/A | 96.6 | 3.4 | 835 | 29 | p.[=];[Pro367Leu] | Yes | BRAF |  | X |
| Prevalence One | 10 | 89712007 | G | G/T | 94.6 | 5.4 | 558 | 32 | p.[=];[Gly209*] | Yes | PTEN |  | X |
| Prevalence One | 17 | 8784043 | C | C/T | 97.2 | 2.8 | 348 | 10 |  | No | PIK3R5 |  | X |
| Prevalence One | 17 | 8785111 | C | C/T | 97.4 | 2.6 | 563 | 15 | p.[=, =];[Glu765Lys, Glu765Lys] | Yes | PIK3R5 |  | X |
| Prevalence One | 17 | 8790860 | G | G/A | 97.8 | 2.2 | 849 | 19 | p.[=, =];[Ser586Phe, Ser586Phe] | Yes | PIK3R5 |  | X |
| Prevalence One | 17 | 8791980 | A | A/T | 79.8 | 20.2 | 202 | 51 | p.[=, =];[Leu375Gln, Leu375Gln] | Yes | PIK3R5 |  | X |
| Prevalence One | 17 | 8791990 | A | A/G | 89.1 | 10.9 | 179 | 22 | p.[=, =];[Ser372Pro, Ser372Pro] | Yes | PIK3R5 |  | X |
| Prevalence One | 17 | 8792003 | C | C/T | 52.4 | 47.6 | 86 | 78 |  | No | PIK3R5 | X |  |
| Prevalence One | 17 | 8792029 | A | A/G | 51.8 | 48.2 | 87 | 81 |  | No | PIK3R5 | X |  |
| Prevalence One | 17 | 8792093 | G | G/A | 53 | 47 | 88 | 78 |  | No | PIK3R5 | X |  |
| Prevalence One | 17 | 8792514 | G | G/A | 50.7 | 49.3 | 386 | 375 |  | No | PIK3R5 | X |  |
| Prevalence One | 17 | 8809025 | G | G/A | 97.8 | 2.2 | 971 | 22 |  | No | PIK3R5 | X |  |
| Prevalence One | 17 | 8814719 | G | G/A | 97.3 | 2.7 | 365 | 10 |  | No | PIK3R5 | X |  |
| Prevalence One | X | 107976940 | G | G/C | 93.9 | 6.1 | 907 | 59 | p.[=];[His879Asp] | Yes | IRS4 | X |  |
| Prevalence One | X | 107978904 | G | G/A | 98.3 | 1.7 | 4045 | 71 | p.[=];[Ala224Val] | Yes | IRS4 |  | X |
| Prevalence One | X | 107978907 | G | G/A | 99 | 1 | 4090 | 42 | p.[=];[Ala223Val] | Yes | IRS4 |  | X |
| Prevalence One | X | 107978957 | G | G/A | 98.8 | 1.2 | 1708 | 21 |  | No | IRS4 |  | X |
| Prevalence One | X | 107978961 | C | C/T | 98.9 | 1 | 1709 | 17 | p.[=];[Arg205His] | Yes | IRS4 |  | X |
| Prevalence One | X | 107979512 | C | C/T | 91.9 | 8.1 | 810 | 71 |  | No | IRS4 | X |  |
| Prevalence One | X | 107979515 | C | C/T | 90.9 | 9.1 | 799 | 80 |  | No | IRS4 | X |  |
| Prevalence One | X | 107979516 | G | G/T | 98.8 | 1.2 | 859 | 10 | p.[=];[Ala20Glu] | Yes | IRS4 |  | X |
| Prevalence Two | 1 | 11181327 | C | C/T | 82.2 | 17.8 | 461 | 100 |  | No | MTOR | X |  |
| Prevalence Two | 1 | 11189815 | G | G/A | 96.2 | 3.6 | 510 | 19 |  | No | MTOR |  | X |
| Prevalence Two | 1 | 11190646 | G | G/A | 89.1 | 10.9 | 179 | 22 |  | No | MTOR | X |  |
| Prevalence Two | 1 | 11205058 | C | T/C | 82.2 | 17.8 | 373 | 81 |  | No | MTOR | X |  |
| Prevalence Two | 1 | 11217226 | G | G/A | 97.3 | 2.7 | 502 | 14 |  | No | MTOR |  | X |
| Prevalence Two | 1 | 11288758 | G | A/G | 83.4 | 16.6 | 131 | 26 |  | No | MTOR | X |  |
| Prevalence Two | 1 | 11301714 | A | G/A | 76.4 | 23.6 | 363 | 112 |  | No | MTOR | X |  |
| Prevalence Two | 1 | 115256529 | T | T/C | 88.6 | 11.4 | 271 | 35 | p.[=];[Gln61Arg] | Yes | NRAS |  | X |
| Prevalence Two | 1 | 115258745 | C | C/G | 96.3 | 3.7 | 591 | 23 | p.[=];[Gly13Arg] | Yes | NRAS |  | X |
| Prevalence Two | 2 | 233990501 | G | G/C | 98.3 | 1.7 | 1281 | 22 |  | No | SHIP1 |  | X |
| Prevalence Two | 2 | 233990594 | A | A/G | 97.8 | 2.2 | 1274 | 28 |  | No | SHIP1 | X |  |
| Prevalence Two | 2 | 234073024 | C | C/T | 98 | 2 | 1298 | 27 | p.[=, =];[Pro257Ser, Pro257Ser] | Yes | SHIP1 |  | X |
| Prevalence Two | 2 | 234093058 | C | C/T | 97.4 | 2.6 | 1140 | 30 | p.[=, =];[Arg476Trp, Arg476Trp] | Yes | SHIP1 |  | X |
| Prevalence Two | 2 | 234113057 | G | G/A | 74.2 | 25.8 | 1261 | 439 | p.[=, =];[Val841Met, Val841Met] | Yes | SHIP1 | X |  |
| Prevalence Two | 2 | 234113301 | C | C/T | 69.6 | 30.4 | 689 | 301 | p.[=, =];[Ala922Val, Ala922Val] | Yes | SHIP1 | X |  |
| Prevalence Two | 3 | 130422592 | T | T/A | 94.6 | 5.4 | 559 | 32 | p.[=];[Met1025Leu] | Yes | PIK3R4 | X |  |
| Prevalence Two | 3 | 130449222 | A | A/G | 88.5 | 11.5 | 1474 | 192 |  | No | PIK3R4 | X |  |
| Prevalence Two | 3 | 178916890 | C | C/T | 89.3 | 10.7 | 151 | 18 | p.[=];[Arg93Trp] | Yes | PIK3CA |  | X |
| Prevalence Two | 3 | 178951998 | A | A/G | 98.9 | 1.1 | 886 | 10 | p.[=];[Asp1018Gly] | Yes | PIK3CA |  | X |
| Prevalence Two | 4 | 55564679 | C | C/T | 98.1 | 1.9 | 504 | 10 |  | No | KIT |  | X |
| Prevalence Two | 4 | 55593464 | A | A/C | 89.7 | 10.3 | 435 | 50 | p.[=, =];[Met537Leu, Met541Leu] | Yes | KIT | X |  |
| Prevalence Two | 4 | 55599268 | C | C/T | 95.8 | 4.2 | 790 | 35 |  | No | KIT | X |  |
| Prevalence Two | 4 | 55602765 | G | G/C | 93.4 | 6.3 | 369 | 25 |  | No | KIT | X |  |
| Prevalence Two | 4 | 103451019 | T | T/C | 71.1 | 28.9 | 3749 | 1523 |  | No | NFKB1 |  | X |
| Prevalence Two | 4 | 103505961 | C | C/T | 96.6 | 3.4 | 281 | 10 |  | No | NFKB1 | X |  |
| Prevalence Two | 4 | 103514658 | T | C/T | 94.2 | 5.6 | 1156 | 69 |  | No | NFKB1 | X |  |
| Prevalence Two | 4 | 103528406 | C | C/T | 98.6 | 1.4 | 1674 | 23 | p.[=, =];[Ser685Phe, Ser684Phe] | Yes | NFKB1 |  | X |
| Prevalence Two | 5 | 67522722 | C | C/T | 90.5 | 9.5 | 268 | 28 |  | No | PIK3R1 | X |  |
| Prevalence Two | 5 | 67569803 | C | C/T | 98.8 | 1.2 | 2215 | 26 | p.[=];[Ser155Phe] | Yes | PIK3R1 |  | X |
| Prevalence Two | 5 | 67586561 | A | A/T | 98 | 1.9 | 1666 | 33 | p.[=];[Tyr2Phe] | Yes | PIK3R1 |  | X |
| Prevalence Two | 5 | 67588148 | G | G/A | 89.5 | 10.5 | 1411 | 165 | p.[=, =, =];[Met326Ile, Met26Ile, Met56Ile] | Yes | PIK3R1 | X |  |
| Prevalence Two | 5 | 67590407 | C | C/T | 96 | 4 | 945 | 39 | p.[=, =, =];[Thr490Ile, Thr190Ile, Thr220Ile] | Yes | PIK3R1 |  | X |
| Prevalence Two | 7 | 140453135 | C | C/T | 94.3 | 5.7 | 1277 | 77 |  | No | BRAF |  | X |
| Prevalence Two | 7 | 140453136 | A | T/A | 57.7 | 42.1 | 727 | 530 | p.[Val600Glu];[=] | Yes | BRAF |  | X |
| Prevalence Two | 7 | 140534494 | T | T/C | 94.6 | 5.4 | 598 | 34 | p.[=];[Asn140Ser] | Yes | BRAF |  | X |
| Prevalence Two | 17 | 8790431 | G | G/A | 98.7 | 1.3 | 1254 | 17 |  | No | PIK3R5 |  | X |
| Prevalence Two | 17 | 8791980 | A | A/T | 86.2 | 13.8 | 237 | 38 | p.[=, =];[Leu375Gln, Leu375Gln] | Yes | PIK3R5 |  | X |
| Prevalence Two | 17 | 8791990 | A | A/G | 94.5 | 5.5 | 224 | 13 | p.[=, =];[Ser372Pro, Ser372Pro] | Yes | PIK3R5 |  | X |
| Prevalence Two | 17 | 8792003 | C | C/T | 61.7 | 38.3 | 127 | 79 |  | No | PIK3R5 | X |  |
| Prevalence Two | 17 | 8792029 | A | A/G | 51.5 | 48.5 | 106 | 100 |  | No | PIK3R5 | X |  |
| Prevalence Two | 17 | 8792093 | G | G/A | 61.3 | 38.7 | 125 | 79 |  | No | PIK3R5 | X |  |
| Prevalence Two | 17 | 8792514 | G | G/A | 54.1 | 45.8 | 506 | 428 |  | No | PIK3R5 | X |  |
| Prevalence Two | 17 | 8809025 | G | G/A | 94.4 | 5.6 | 747 | 44 |  | No | PIK3R5 | X |  |
| Prevalence Two | X | 107975967 | G | G/A | 96.8 | 3.2 | 701 | 23 | p.[=];[Ala1203Val] | Yes | IRS4 |  | X |
| Prevalence Two | X | 107975968 | C | C/T | 96.8 | 3 | 701 | 22 | p.[=];[Ala1203Thr] | Yes | IRS4 |  | X |
| Prevalence Two | X | 107976357 | C | C/T | 97.6 | 2.3 | 911 | 21 | p.[=];[Arg1073Lys] | Yes | IRS4 |  | X |
| Prevalence Two | X | 107976362 | T | T/A | 97.9 | 2.1 | 913 | 20 |  | No | IRS4 |  | X |
| Prevalence Two | X | 107976940 | G | G/C | 86.4 | 13.6 | 1305 | 206 | p.[=];[His879Asp] | Yes | IRS4 | X |  |
| Prevalence Two | X | 107978342 | T | T/A | 97.4 | 2.6 | 952 | 25 |  | No | IRS4 | X |  |
| Prevalence Two | X | 107978343 | C | C/T | 97.4 | 2.6 | 951 | 25 | p.[=];[Arg411Gln] | Yes | IRS4 | X |  |
| Prevalence Two | X | 107979278 | T | T/G | 97.5 | 2.5 | 809 | 21 | p.[=];[Lys99Asn] | Yes | IRS4 |  | X |
| Prevalence Two | X | 107979279 | T | T/A | 97.5 | 2.5 | 809 | 21 | p.[=];[Lys99Ile] | Yes | IRS4 |  | X |
| Prevalence Two | X | 107979437 | G | G/A | 95.8 | 4.2 | 752 | 33 |  | No | IRS4 | X |  |
| Prevalence Two | X | 107979475 | G | G/A | 96.4 | 3.6 | 728 | 27 | p.[=];[Leu34Phe] | Yes | IRS4 | X |  |
| Prevalence Two | X | 107979512 | C | C/T | 80.3 | 19.7 | 622 | 153 |  | No | IRS4 | X |  |
| Prevalence Two | X | 107979515 | C | C/T | 83.3 | 16.7 | 649 | 130 |  | No | IRS4 | X |  |
